# Risk factors for astigmatic components and internal compensation: the Nanjing Eye Study

**DOI:** 10.1038/s41433-020-0881-5

**Published:** 2020-04-22

**Authors:** Zijin Wang, Haohai Tong, Qingfeng Hao, Xuejuan Chen, Hui Zhu, Dan Huang, Rui Li, Zhibin Hu, Hu Liu

**Affiliations:** 1 Department of Ophthalmology, The First Affiliated Hospital with Nanjing Medical University, 300 Guangzhou Road, 210029 Nanjing, China; 2 State Key Laboratory of Reproductive Medicine, Nanjing Medical University, 101 Longmian Street, 211166 Nanjing, China; 3 Department of Epidemiology and Biostatistics, Jiangsu Key Lab of Cancer Biomarkers, Prevention and Treatment, Collaborative Innovation Center for Cancer Personalized Medicine, School of Public Health, Nanjing Medical University, 101 Longmian Street, 211166 Nanjing, China

**Keywords:** Risk factors, Paediatrics, Epidemiology

## Abstract

**Purpose:**

To determine the risk factors for total astigmatism (TA), anterior corneal astigmatism (ACA), and internal compensation in Chinese preschool children.

**Methods:**

In the population-based Nanjing Eye Study, children were measured for noncycloplegic refractive error and for biometric parameters. Data from questionnaires and measures from right eyes were analyzed for determining risk factors for TA, ACA, and internal compensation from multivariate logistic regression models.

**Results:**

Of 1327 children (66.8 ± 3.4 months, 53.2% male), older age of the child (OR = 0.95 for per month increase; *P* = 0.03), older paternal age at child birth (OR = 1.04 for per year increase; *P* = 0.03), paternal astigmatism (OR = 1.89; *P* = 0.003), maternal astigmatism (OR = 1.73, *P* = 0.008), and second-hand smoke exposure during pregnancy (OR = 1.64; *P* = 0.03) were associated with higher risk of TA, while partial breastfeeding (OR = 0.49, *P* = 0.006) or formula feeding (OR = 0.46, *P* = 0.003) were associated with lower risk of TA. Larger ratio of axial length to corneal radius (OR = 16.16 for per unit increase; *P* = 0.001), maternal working during pregnancy (OR = 1.27; *P* = 0.04), and cesarean delivery (OR = 1.68, *P* = 0.04) were associated with higher risk of ACA, while formula feeding was associated with lower risk of ACA (OR = 0.57, *P* = 0.01). Paternal astigmatism (OR = 0.50, *P* = 0.01) and assisted reproduction (OR = 0.56, *P* = 0.03) were associated with lower risk of horizontal or vertical internal compensation. More outdoor activity time (OR = 1.15 for per hour increase, *P* = 0.01) was associated with higher risk of oblique internal compensation while more nighttime sleep on weekends (OR = 0.83 for per hour increase, *P* = 0.01) was associated with lower risk of oblique internal compensation.

**Conclusions:**

Our study confirmed some previously reported risk factors and identified some novel risk factors for astigmatism including formula feeding for lower risk of both ACA and TA, and older paternal age at child birth for higher risk of TA.

## Introduction

Astigmatism is an important clinical and public health problem; If uncorrected, astigmatism can lead to continuous blurred vision at any distance.

Previous studies have found many factors associated with astigmatism including high risk genes, eyelid pressure, extraocular muscle tension, gestational age, birth weight, nutrition [1], ethnicity, spherical equivalent refractive error, maternal smoking during pregnancy [2], accommodative convergence/accommodation ratio (AC/A) [3], age, education, ametropia [4], the axial length–corneal radius ratio (AL/CR) [5], delivery mode [6], socio-economically deprivation [7] and iris color [8]. In a recent paper on 48–60-month-old Chinese children [9], we reported the prevalence of the components of astigmatism, described the difference between corneal astigmatism (CA) and anterior corneal astigmatism (ACA), and demonstrated the compensatory role of internal astigmatism (IA) in reducing ACA. In addition, we evaluated the role of age, gender, and AL/CR on the components of astigmatism. However, our study did not evaluate the other potential risk factors as these data were not collected. During ongoing Nanjing Eye Study (NES), we collected additional potential risk factors, particularly the factors during pregnancy and early childhood, a critical period for the development of refractive error [10, 11]. Furthermore, previous studies only evaluated risk factors for total astigmatism (TA) and CA. As we already demonstrated the compensatory role of IA, analysis for risk factors of internal compensation may lead to new discoveries and help build the overall framework of astigmatic genesis and development.

This study was to perform a comprehensive evaluation of potential risk factors for TA, ACA, and internal compensation in a large population-based NES.

## Materials and methods

### Study population

The NES is an ongoing population-based open cohort study, designed to longitudinally observe the onset and progression of childhood ocular diseases in eastern China [9, 12–15]. The study was approved by the institutional review board in The First Affiliated Hospital with Nanjing Medical University and was conducted in accordance with the tenets of the Declaration of Helsinki. Written consent was obtained from the parents or guardians of all children. The study population for the present study consisted of 61–72-month-old children enrolled in kindergarten in the Yuhuatai District of Nanjing City in East China and born between September 2011 and August 2012. Data from eye examinations and questionnaire presented in this paper were collected in 2017.

### Eye examination

Six experienced ophthalmologists and four optometrists specialized in pediatric eye care performed comprehensive eye examinations following the standard study protocol. The noncycloplegic refractive status of both eyes of each participant was measured using an autorefractor (Cannon RF10; Canon, Tokyo, Japan). The optic low-coherent reflectometer (LenStar LS-900; Haag-Streit AG, Koeniz, Switzerland) was used to obtain biometric parameters, including corneal curvatures and axial length. The examining procedure has been described in detail previously [9].

A comprehensive questionnaire was distributed to legal guardians of each child. The questionnaire collected data including basic information of children and parents, history of pregnancy, birth, feeding status, daily activities within the past year, sleep quality within the past month, home environment, and guardian’s concerns over eyes. We calculated average daily hours spent on activities classified as near-work activities, mid-working distance activities, and outdoor activities. The 5-min Apgar score at birth was categorized as normal (≥7) and abnormal (<7).

### Definition

Astigmatism was defined as a cylinder magnitude worse than or equal to 1.0 diopter (D), expressed as a negative cylinder form. The magnitude of ACA was calculated as the difference between the flattest and steepest corneal meridians of the anterior corneal surface with the cylindrical axis equal to the flattest meridian. The magnitude of IA was calculated as the vectorial difference between TA and ACA. The presence of TA was defined as TA equal to −1 or less, and the presence of ACA was defined as ACA equal to −1 or less. To decompose TA and ACA, the vector method modified by Thibos [16] was used for the following calculations:

$${\it{SE\,=\,S\,+\,C/}}{2},$$

$${\it{J}}_{{0}}{\it{\,=\,}}\left( {{\it{ - C/}}}{2} \right){\it{\,\times\,(cos}}\;2{\it{A)}},$$

$${\it{J}}_{{{45}}}{\it{\,=\,}}\left( {{\it{ - C/}}}2 \right){\it{\,\times\,(sin}}\;2{\it{A)}},$$

where *SE* is the spherical equivalent, *S* is sphere, *C* is the cylinder in minus format, *A* is the cylinder axis, *J*_0_ and *J*_45_ are the horizontal or vertical and oblique vectors of the cylinder, respectively. According to these methods, *J*_0t_*, J*_0a_*, and J*_0i_ were calculated, representing *J*_0_ of TA, ACA, and IA respectively. Similar calculations were made for *J*_45t_*, J*_45a_*, and J*_45i_.

The compensation factor (CF) was calculated as the negative ratio of IA and ACA. *J*_0_ and *J*_45_ were used to evaluate CF as following:

$${\it{CF}}_{{0}}{\it{\,=\,- J}}_{{0\it{i}}}{\it{/J}}_{{0\it{a}}},$$

$${\it{CF}}_{{{45}}}{\it{\,=\,- J}}_{{45\it{i}}}{\it{/J}}_{{45\it{a}}}.$$

The compensation types were classified as following based on the calculated CF [9, 17, 18]. The presence of internal compensation was defined as CF 0.1–2. If CF < 0.1 or CF > 2, it was categorized as without internal compensation.

AL/CR was calculated as the axial length (mm) divided by the mean radius of curvature (mm).

### Statistical analysis

The Statistical Package for the Social Sciences (SPSS V.13.0; IBM, Chicago, IL, USA) was used for all statistical analyses. Results were presented as mean ± standard deviation (SD) for continuous variables, as percentage for categorical variables. Characteristics of children included in the analysis were compared with those excluded due to missing data in questionnaire using two-sample *t*-test for comparisons of means and chi-square test for comparison of proportions. Comparisons of distribution of each candidate risk factor were performed between children with vs. without TA, between children with vs. without ACA, and between children with vs. without internal compensation using two-sample *t*-tests for continuous variables and chi-square tests for categorical variables. Multivariate logistic regression models using forward variable selection based on likelihood ratio test were performed to determine statistically significant risk factors for TA, ACA, CF_0_, and CF_45_. Odds ratios (OR) and their 95% confidence intervals (95% CI) were calculated from multivariate logistic regression models. All statistical tests were two-sided and *P* < 0.05 was considered statistically significant.

## Results

### Characteristics of study population

Among 2300 eligible preschoolers, 1920 (participation rate 83.5%) children completed comprehensive eye examinations. Among these 1920 children, 138 children were uncooperative and no refraction measurements or biometric parameters from right eye were obtained after several attempts. Guardians of 455 children did not complete the questionnaire, leaving 1327 children (69.1% of eligible participants) included in this study. Their mean (±SD) age was 66.8 ± 3.4 months and 706 (53.2%) of participants were boys.

There were no significant differences in characteristics of children (including age, gender, prevalence rate of TA and ACA from right eyes) between children included in the analysis and those excluded from analysis due to missing data in questionnaire.

### Prevalence and compensation condition of astigmatism

The prevalence rate was 12.9% (171/1327) for TA and 64.4% (854/1327) for ACA. CF analysis revealed that the compensation rate was 94.0% (1247/1327) in *J*_0_, and 74.1% (983/1327) in *J*_45_.

### **Risk factors**

We evaluated a total of 25 risk factors (Table 1) for TA, ACA, CF_0_, and CF_45_.

**Table 1:** Distribution of risk factors in children with and without total astigmatism.

Risk Factors	Without total astigmatism (*n* = 1156)	With total astigmatism (*n* = 171)	*P* value
Mean (±SD) age (month)	66.92 ± 3.42	66.32 ± 3.11	0.02
Mean (±SD) paternal age at child birth (year)	27.80 ± 4.74	28.73 ± 4.98	0.02
Mean (±SD) maternal age at child birth (year)	26.16 ± 3.94	26.74 ± 3.73	0.06
Mean (±SD) birth weight (kilogram)	3.33 ± 0.53	3.32 ± 0.50	0.79
Mean (±SD) near-work activity (hour)	4.76 ± 3.36	4.80 ± 4.80	0.90
Mean (±SD) mid-working distance activity (hour)	1.54 ± 1.59	1.40 ± 2.07	0.38
Mean (±SD) outdoor activity (hour)	2.21 ± 1.30	2.34 ± 1.70	0.33
Mean (±SD) average nighttime sleep on weekdays (hour)	9.91 ± 0.67	9.82 ± 0.67	0.10
Mean (±SD) average nighttime sleep on weekends (hour)	10.21 ± 0.86	10.10 ± 0.85	0.12
AL/CR^a^	2.87 ± 0.06	2.88 ± 0.08	0.07
Gender: male (%)	611 (52.9%)	95 (55.6%)	0.51
Paternal myopia: yes(%)	411 (35.6%)	68 (39.8%)	0.28
Maternal myopia: yes (%)	449 (38.8%)	80 (46.8%)	0.048
Paternal astigmatism yes (%)	145 (12.5%)	40 (23.4%)	<0.001
Maternal astigmatism: yes (%)	169 (14.6%)	43 (25.1%)	<0.001
Mode of pregnancy: assisted (%)	188 (16.3%)	37 (21.6%)	0.08
Term delivery (full-term:pre-term:post-term)			0.28
Full-term	1042 (90.1%)	160 (93.6%)	
Pre-term	58 (5.0%)	7 (4.1%)	
Post-term	56 (4.8%)	4 (2.3%)	
5-min Apgar score: Abnormal (%)	31(2.7%)	9(5.3%)	0.07
Delivery mode			0.64
Vaginal	655 (56.7%)	94 (55.0%)	
Vaginal transferring to cesarean	91 (7.9%)	11 (6.4%)	
Cesarean	410 (35.5%)	66 (38.6%)	
Oxygen uptake after birth: yes (%)	71 (6.14%)	8 (4.7%)	0.45
Second or third child: yes (%)	219 (18.9%)	39 (22.8%)	0.23
Twin or triple: yes (%)	25 (2.2%)	6 (3.5%)	0.41
Feeding patterns			0.02
Exclusive breastfeeding	561 (48.5%)	78 (45.6%)	
Partial breastfeeding	493 (42.6%)	66 (38.6%)	
Formula feeding	102 (8.8%)	27 (15.8%)	
Maternal working during pregnancy: yes (%)	522 (45.2%)	92 (53.8%)	0.03
Second-hand smoke exposure during pregnancy: yes (%)	143 (12.4%)	32 (18.7%)	0.02

^a^AL/CR: ratio of axial length to corneal radius.

Comparisons for each risk factor between children with and without TA are shown in Table 1. Children with TA were more likely to be younger (*P* = 0.02), have older father at child birth (*P* = 0.02), more likely to have myopic mothers (*P* = 0.048), astigmatic mothers or astigmatic fathers (both *P* < 0.001) than children without TA. In the children with TA, percentage of maternal working during pregnancy (*P* = 0.03) and second-hand smoke exposure during pregnancy (*P* = 0.02) were higher than children without TA. Children with TA were also more likely to be fed with breast milk than children without TA (*P* = 0.02).

In the multivariate analysis, six risk factors were significantly independently associated with TA (Table 2): age of the child, paternal age at child birth, paternal astigmatism, maternal astigmatism, second-hand smoke exposure during pregnancy, and feeding patterns. Older age of the child was significantly associated with lower risk of TA (OR = 0.95 for per month increase; 95% CI 0.90, 0.996), while older paternal age at child birth was associated with higher risk of TA (OR = 1.04 for per year increase; 95% CI 1.004, 1.07). Children with astigmatic fathers were 1.89 times as likely to have TA as children with non-astigmatic fathers (95% CI 1.25, 2.86). Children with astigmatic mothers were 1.73 times as likely to have TA as children with non-astigmatic mothers (95% CI 1.16, 2.59). Children whose mothers had second-hand smoke exposure during pregnancy were 1.64 times as likely to have TA as children whose mothers did not have second-hand smoke exposure (95% CI 1.06, 2.54). Concerning feeding patterns, children with partial breastfeeding were 0.50 times as likely to have TA as children with exclusive breastfeeding (95% CI 0.30, 0.82), while children with formula feeding were 0.46 times as likely to have TA as children with exclusive breastfeeding (95% CI 0.28, 0.77).

**Table 2:** Independent risk factors for total astigmatism from multivariate logistic regression.

Risk factors	Multivariate analysis		
	Adjusted OR	95% CI	*P*
Month age of the child (per month increase)	0.95	0.90–0.996	0.03
Paternal age at child birth (per year increase)	1.04	1.004–1.07	0.03
Paternal astigmatism (yes vs. no)	1.89	1.25–2.86	0.003
Maternal astigmatism (yes vs. no)	1.73	1.16–2.59	0.008
Second-hand smoke exposure during pregnancy (yes vs. no)	1.64	1.06–2.54	0.03
Feeding patterns			
Exclusive breastfeeding	Reference		
Partial breastfeeding	0.50	0.30–0.82	0.006
Formula feeding	0.46	0.28–0.77	0.003

*OR* odds ratio, *CI* confidence interval.

Comparisons for each risk factor were performed between children with and without ACA (sTable 1). Children with ACA were more likely to have larger AL/CR than children without ACA (*P* = 0.002). There was significant difference between children with ACA and without ACA in delivery mode (*P* = 0.02) and feeding patterns (*P* = 0.02). The proportion of oxygen uptake after birth was higher in the children with ACA (*P* = 0.02).

In the multivariate analysis, four variables were significantly associated with ACA (Table 3): AL/CR, maternal working during pregnancy, feeding patterns, and delivery mode. Larger AL/CR was significantly associated with higher risk of ACA (OR = 16.63 per unit increase; 95% CI 3.03, 91.24). Children whose mothers working during pregnancy were 1.26 times as likely to have ACA as children whose mother stopped working during pregnancy (95% CI 1.01, 1.59). Concerning feeding patterns, ACA was not significantly different between children with partial breastfeeding and children with exclusive breastfeeding, while children with formula feeding were 0.57 times as likely to have ACA as children with exclusive breastfeeding (95% CI 0.37, 0.88). Concerning delivery mode, ACA was not significantly different between children born by vaginal delivery transferring to cesarean delivery and children born by vaginal delivery, while children born by cesarean delivery were 1.67 times as likely to have ACA as children born by vaginal delivery (95% CI 1.02, 2.74).

**Table 3:** Independent risk factors for anterior corneal astigmatism from multivariate logistic regression.

	Multivariate analysis		
Risk factors	Adjusted OR	95% CI	*P*
AL/CR (per unit increase)	16.63	3.03–91.24	0.001
Maternal work during pregnancy (yes vs. no)	1.26	1.01–1.59	0.046
Feeding patterns			
Exclusive breastfeeding	Reference group		
Partial breastfeeding	0.75	0.49–1.15	0.19
Formula feeding	0.57	0.37–0.88	0.01
Delivery mode			
Vaginal delivery	Reference		
Vaginal delivery transferring to cesarean delivery	0.86	0.67–1.09	0.21
Cesarean delivery	1.67	1.02–2.74	0.04

*OR* odds ratio, *CI* confidence interval.

Comparisons for each risk factor were performed between children with and without internal compensation (sTables 2 and  3). Children with horizontal or vertical internal compensation were more likely to have astigmatic fathers than children without horizontal or vertical internal compensation (*P* = 0.009). The percentage of assisted reproduction was lower in the children with horizontal or vertical internal compensation (*P* = 0.02). Mean outdoor activity time of children with oblique internal compensation was more than children without oblique internal compensation (*P* = 0.002). Mean average nighttime sleep on weekends was slightly less in children with oblique internal compensation than children without oblique internal compensation (*P* = 0.03). Mean average nighttime sleep on weekends was slightly less in children with oblique internal compensation than children without oblique internal compensation (*P* = 0.03).

In the multivariate analysis, paternal astigmatism and mode of pregnancy were significantly associated with horizontal or vertical internal compensation, while outdoor activity and sleep duration at night on weekends were significantly associated with oblique internal compensation (Table 4). Children with astigmatic fathers were 0.50 times as likely to have horizontal or vertical internal compensation as children with non-astigmatic father (95% CI 0.29, 0.87). Children born by assisted reproduction were 0.56 times as likely to have horizontal or vertical internal compensation as children born by spontaneous conception (95% CI 0.33, 0.95). More outdoor activity time (OR = 1.15 for per hour increase, 95% CI 1.03, 1.29) was associated with higher risk of oblique internal compensation while more nighttime sleep on weekends was associated with lower risk of oblique internal compensation (OR = 0.84 for per hour increase, 95% CI 0.72, 0,97).

**Table 4:** Independent risk factors for internal compensation from multivariate logistic regression.

	Multivariate analysis		
Risk factors	Adjusted OR	95% CI	*P*
**CF0**			
Paternal astigmatism (yes vs. no)	0.50	0.29–0.87	0.01
Mode of pregnancy (spontaneous vs. assisted)	0.56	0.33–0.95	0.03
**CF45**			
Outdoor activity (per hour increase)	1.15	1.03–1.29	0.01
Sleep duration at night on weekends (per hour increase)	0.84	0.72–0.97	0.02

*OR* odds ratio, *CI* confidence interval.

## Discussion

Although previous studies have described the characteristics of children with astigmatism, these studies only focused on the risk factors for TA or CA. Few studies assessed risk factors for astigmatism by integrating the components of astigmatism and taking internal compensation into consideration. This study explored the risk factors of TA, ACA, and internal compensation in preschoolers. These risk factors included characteristics during pregnancy and early childhood, which were rarely studied previously for astigmatism, but important to investigate because pregnancy to early childhood is the critical time window for the development of refraction and were more analyzed in the spherical equivalent refraction before.

This study found that older age of the child was significantly associated with lower risk of TA but not associated with ACA. Most astigmatism, especially with-the-rule astigmatism, showed a trend of decreasing with age, and reached stable at 18–24 months [10, 11, 19, 20]. CA tended to decrease between the age of 3 years and 8 years, but the decrease was generally small and would not be considered clinically significant [21].

This study found older paternal age at child birth was associated with higher risk of TA, which has not been reported in the literature as we know. The exact mechanism for this association is not clear. However, older paternal age at child birth has been reported to be associated with some adverse medical conditions including stillbirths, musculo-skeletal syndromes, cleft palate, acute lymphoblastic leukemia, retinoblastoma, and neurodevelopmental disorders in the autism spectrum and schizophrenia [22]. Biologically, there existed nonlinear increase in germ-line mutations in males with older age [23]. Social effects could also be reasons for this association, such as the different way older fathers interact with children [24]. Further study is needed to confirm this association.

This study revealed that both paternal astigmatism and maternal astigmatism were risk factors of TA, but they were not associated with ACA. Paternal astigmatism was also associated with lower likelihood of horizontal or vertical internal compensation. Previous genetic studies on astigmatism provided contradicting results on the genetic contribution to astigmatism. Wixson concluded that both parents seemed to play roles in determining the corneal power characteristics of the child [25]. Early twin studies showed that the correlations between monozygotic twins for astigmatism were not significantly different from the correlations between dizygotic twins, which suggested that the genetic contribution to astigmatism was low [26–28]. Lee et al. found minimal association existed among family members for astigmatism [29]. However, some other twin studies suggested a genetic etiology in astigmatism development, with the estimated heritability ranging from 30 to 60% [30–34]. A meta-analysis of five Asian cohorts identifies PDGFRA as a susceptibility locus for CA [35]. Further genetic studies are needed to determine whether genetic etiology plays a role in ACA or internal compensation, which may affect the development of TA.

This study found that second-hand smoke exposure during pregnancy was associated with high risk of TA. The multi-ethnic pediatric eye disease study (MEPEDS) showed that preschool children whose mothers smoked during pregnancy were 1.5 times as likely to have astigmatism as children whose mothers did not smoke [2]. Mothers who smoked during pregnancy accounted for 8.9% in the MEPEDS, while no mother smoked during pregnancy in our study. However, in our study, 8.9% mothers had second-hand smoke intake during pregnancy. Fetal exposure to maternal smoking is associated with numerous adverse developmental consequences during intrauterine and postnatal period. Certain biologically active substances in cigarette products, such as nicotine, cyanide, and carbon monoxide, can cross the placental barrier and induce fetal hypoxia [36]. A systematic review showed maternal smoking during pregnancy was associated with an increased risk of visual disturbances including refractive errors during childhood [37]. Both active and passive maternal smoking during pregnancy may have adverse effect on refractive error development in a dose-dependent manner [37].

Interestingly, this study showed that formula feeding reduced the risk of both TA and ACA compared with exclusive breastfeeding. Furthermore, partial breastfeeding also reduced the risk of TA compared with exclusive breastfeeding. As far as we know, none of previous studies explored the relationship between feeding patterns and astigmatism. Some previous studies suggested that infants with breastfeeding will have better vision and be less likely to be myopic in later life than those with formula feeding [38, 39]. A study in young Singapore Chinese children showed that breastfeeding was associated with more positive spherical equivalent refraction [40]. However, another two studies did not find any significant relationship between feeding patterns and visual acuity and spherical equivalent refraction [41, 42]. Future investigations are needed to validate our finding and determine what difference between breast milk and formula might affect the development of cornea.

This study found that larger AL/CR increased the risk of ACA, but was not associated with TA. The association between AL/CR and ACA is consistent with a previous study in school children [5], and it was hypothesized that the lengthening of the AL and the shortening of the CR both could increase the pressure of the eyelids on the eye globes and ultimately lead to a corneal deformation [5].

This study revealed that cesarean section increased the risk of ACA compared with vaginal delivery, but the delivery mode was not associated with both TA and internal compensation. A previous study showed that children born by cesarean section, especially those born by elective cesarean section, were more likely to have severe TA ( ≤ −2.50 D) compared with children born by vaginal delivery [6]. Another study revealed that at birth, neonates delivered vaginally had a greater frequency of with-the-rule CA than those delivered by cesarean section [43]. These results may be because infants delivered by cesarean section experience less or no contractions of the uterus compared with those delivered naturally. The pressure from the uterus and birth canal may contribute to changes in corneal curvature. The changes could also be explained by different levels of exposure from hormonal milieu of labor [6].

This study showed that children through assisted reproduction were less likely to have horizontal or vertical internal compensation than children through spontaneous conception, but the pregnancy mode was not associated with both TA and ACA. Due to special genetic background and environmental conditions, infants through assisted reproduction may be different from naturally conceived infants [44]. A study in infants born by assisted reproduction showed higher TA prevalence [44]. However, Axer-Siegel et al. found that in vitro fertilization did not affect the incidence of CA compared with spontaneous conception [45]. A study by Wikstrand did not show any significant difference in TA between children born after intra-cytoplasmic sperm injection and children born by spontaneous conception [46].

This study found that more outdoor activity time increased the likelihood of oblique internal compensation. It has been well known that outdoor activity reduces the risk of myopia [47]. Studies have found that children with myopia or hyperopia were more likely to have astigmatism than children without spherical refractive error [2, 48]. Therefore, the association between outdoor activity and astigmatic internal compensation seems reasonable. We can infer that oblique internal compensation may reduce the risk of oblique TA. Moreover, it is easier to cause amblyopia with a small degree of initial oblique astigmatism than orthogonal astigmatism [49, 50].

This study showed less nighttime sleep on weekends was associated with higher likelihood of oblique internal compensation. The exact mechanism for this association is unknown. Thus, future study using more precise sleep monitoring methods is needed to confirm this association.

The risk factors of TA and ACA are not completely consistent, so we can speculate that not all factors that produce change of ACA will directly lead to the change of TA. TA is not solely determined by ACA or IA, while the disturbance of internal compensation may be a possible way to cause the change of TA. The risk factors of TA, ACA, and internal compensation are different, likely due to the following reasons. First, astigmatism is a vector and vectorial decomposition may interfere with the analysis of astigmatic risk factors, but there is no better method to replace this. Second, the components of astigmatism interact with each other, so that the risk factors of astigmatic components may enhance, weaken, or offset the function of each other. Their exact mechanisms and their interactions need further study.

The strengths of this study include its population-based design, large sample size, and standardized examination protocols performed by an expert team. The age range of the subjects is relatively narrow, eliminating the impact of large age span on TA and ACA. Our analyses of risk factors for astigmatism are different from previous studies, by considering the correlation of components and internal compensation. The limitation of this study is that some eligible children were not included into the analysis due to missing data in questionnaire or refractive error measures. In addition, the risk factor data collected through questionnaire may be subjective and biased.

In summary, this study confirmed several previously reported risk factors and identified several novel risk factors for astigmatism. We found maternal astigmatism and second-hand smoke exposure during pregnancy were independent risk factors for TA; higher AL/CR, cesarean section were independent risk factors for ACA; assisted reproduction was an independent risk factor for horizontal or vertical compensation; and less outdoor activity time and more nighttime sleep on weekends were independent risk factors for oblique compensation. Compared with exclusive breastfeeding, formula feeding was associated with reduced risk in both ACA and TA. Paternal astigmatism was associated with lower risk of horizontal or vertical internal compensation and higher risk of TA. Although the novel risk factors need further validation in future studies, these new findings may help us understand the development of astigmatism and develop strategies to prevent or treat astigmatism in children.

## Summary

### What was known before

- Previous studies have found many factors associated with astigmatism.
- Previous studies only evaluated risk factors for total astigmatism and corneal astigmatism. Few studies assessed risk factors for astigmatism by integrating the components of astigmatism and taking internal compensation into consideration.
- Risk factors enrolled before lack characteristics during pregnancy and early childhood.

### What this study adds

- Our previous study already demonstrated the compensatory role of internal astigmatism. This study explored the risk factors of total astigmatism, anterior corneal astigmatism, and internal compensation in preschoolers.
- We collected risk factors during pregnancy and early childhood.
- This study confirmed several previously reported risk factors and identified several novel risk factors for astigmatism including formula feeding for lower risk of both anterior corneal astigmatism and total astigmatism, and older paternal age at child birth for higher risk of total astigmatism.

## Supplementary information

sTable 1

sTable 2

sTable 3
