# Supplementary material for: Risk factors for astigmatic components and internal compensation: the Nanjing Eye Study
Source: Eye (Lond). 2020 Apr 22;35(2):499–507. doi: 10.1038/s41433-020-0881-5 (PMC8026993; doi:10.1038/s41433-020-0881-5)
Supplement: Supplementary file 1 — sTable 1 [file 41433_2020_881_MOESM1_ESM.docx]

**sTable 1. Distribution of Risk Factors in Children With and Without Anterior Corneal Astigmatism**

| **Risk Factors** | **Without Anterior Corneal Astigmatism (N = 473)** | **With Anterior Corneal Astigmatism (N = 854)** | ***P*-value** |
| --- | --- | --- | --- |
| Mean (± SD) age (month) | 67.05±3.44 | 66.72±3.36 | 0.099 |
| Mean (± SD) paternal age at child birth (year) | 27.66±4.63 | 28.06±4.86 | 0.14 |
| Mean (± SD) maternal age at child birth (year) | 25.96±3.87 | 26.38±3.94 | 0.06 |
| Mean (± SD) birth weight (kilogram) | 3.35±0.50 | 3.32±0.54 | 0.28 |
| Mean (± SD) near-work activity (hour) | 4.60±3.06 | 4.85±3.83 | 0.18 |
| Mean (± SD) mid-working distance activity (hour) | 1.54±1.55 | 1.52±1.72 | 0.76 |
| Mean (± SD) outdoor activity (hour) | 2.26±1.35 | 2.20±1.36 | 0.43 |
| Mean (± SD) average nighttime sleep on weekdays (hour) | 9.88±0.64 | 9.91±0.68 | 0.44 |
| Mean (± SD) average nighttime sleep on weekends (hour) | 10.18±0.75 | 10.21±0.91 | 0.57 |
| †AL/CR | 2.87±0.06 | 2.88±0.07 | 0.002 |
| Gender: male (%) | 260(55.0%) | 446(52.2%) | 0.34 |
| Paternal myopia: yes(%) | 173(36.6%) | 306(35.8%) | 0.79 |
| Maternal myopia:yes (%) | 188(39.7%) | 341(39.9%) | 0.95 |
| Paternal astigmatism yes (%) | 59(12.5%) | 126(14.8%) | 0.25 |
| Maternal astigmatism: yes (%) | 69(14.6%) | 143(16.7%) | 0.30 |
| Mode of pregnancy: assisted (%) | 78(16.5%) | 147(17.2%) | 0.74 |
| Term delivery |  |  | 0.61 |
| Full-term | 425(89.8%) | 777(91.0%) |  |
| Pre-term | 23(4.9%) | 42(4.9%) |  |
| Post-term | 25(5.3%) | 35(4.1%) |  |
| 5-min Apgar score: Abnormal (%) | 16(3.4%) | 24(2.8%) | 0.56 |
| Delivery mode |  |  | 0.02 |
| Vaginal | 284(60.0%) | 465(54.5%) |  |
| Vaginal transferring to cesarean | 25(5.3%) | 77(9.0%) |  |
| Casarean | 164(34.7%) | 312(36.5%) |  |
| Oxygen uptake after birth: yes (%) | 20(4.2%) | 59(6.9%) | 0.048 |
| Second or third child: yes (%) | 83(17.5%) | 175(20.5%) | 0.19 |
| Twin or triple: yes (%) | 9(1.9%) | 22(2.6%) | 0.44 |
| Feeding patterns |  |  | 0.02 |
| Exclusive breastfeeding | 217(45.9%) | 422(49.4%) |  |
| Partial breastfeeding | 220(46.5%) | 339(39.7%) |  |
| Formula feeding | 36(7.6%) | 93(10.9%) |  |
| Maternal working during pregnancy: yes (%) | 205(43.3%) | 409(47.9%) | 0.11 |
| Second-hand smoke exposure during pregnancy: yes (%) | 53(11.2%) | 122(14.3%) | 0.11 |

† AL/CR: ratio of axial length to corneal radius
