# Supplementary material for: Risk factors for astigmatic components and internal compensation: the Nanjing Eye Study
Source: Eye (Lond). 2020 Apr 22;35(2):499–507. doi: 10.1038/s41433-020-0881-5 (PMC8026993; doi:10.1038/s41433-020-0881-5)
Supplement: Supplementary file 2 — sTable 2 [file 41433_2020_881_MOESM2_ESM.docx]

**sTable 2. Distribution of Risk Factors in Children With and Without Horizontal or Vertical Internal Compensation**

| **Risk Factors** | **Without Horizontal or Vertical Internal Compensation (CF_0_) (N = 80)** | **With Horizontal or Vertical Internal Compensation (CF_0_) (N = 1247)** | ***P*-value** |
| --- | --- | --- | --- |
| Mean (± SD) age (month) | 66.83±3.31 | 66.84±3.40 | 0.97 |
| Mean (± SD) paternal age at child birth (year) | 27.79±4.10 | 28.92±4.82 | 0.78 |
| Mean (± SD) maternal age at child birth (year) | 26.09±3.30 | 26.24±3.96 | 0.69 |
| Mean (± SD) birth weight (kilogram) | 3.26±0.51 | 3.34±0.53 | 0.20 |
| Mean (± SD) near-work activity (hour) | 5.35±6.67 | 4.72±3.28 | 0.40 |
| Mean (± SD) mid-working distance activity (hour) | 1.63±2.82 | 1.52±1.56 | 0.72 |
| Mean (± SD) outdoor activity (hour) | 2.46±1.90 | 2.21±1.31 | 0.25 |
| Mean (± SD) average nighttime sleep on weekdays (hour) | 9.86±0.63 | 9.90±0.67 | 0.64 |
| Mean (± SD) average nighttime sleep on weekends (hour) | 10.25±0.78 | 10.19±0.86 | 0.49 |
| †AL/CR | 2.88±0.07 | 2.87±0.07 | 0.59 |
| Gender: male (%) | 44(55.0%) | 662(53.1%) | 0.74 |
| Paternal myopia: yes(%) | 32(40.0%) | 447(35.8%) | 0.45 |
| Maternal myopia:yes (%) | 32(40.0%) | 497(39.9%) | 0.98 |
| Paternal astigmatism yes (%) | 19(23.7%) | 166(13.3%) | 0.009 |
| Maternal astigmatism: yes (%) | 18(22.5%) | 194(15.6%) | 0.10 |
| Mode of pregnancy: assisted (%) | 21(26.2%) | 204(16.4%) | 0.02 |
| Term delivery: pre-term or post-term (%) | 7(8.7%) | 118(9.5%) | 0.83 |
| 5-min Apgar score: Abnormal (%) | 4(5%) | 36(2.9%) | 0.46 |
| Delivery mode |  |  | 0.38 |
| Vaginal | 48(60.0%) | 701(56.2%) |  |
| Vaginal transferring to cesarean | 3(3.8%) | 99(7.9%) |  |
| Casarean | 29(36.2%) | 447(35.9%) |  |
| Oxygen uptake after birth: yes (%) | 5(6.2%) | 74(5.9%) | 1.00 |
| Second or third child: yes (%) | 16(20.0%) | 242(19.4%) | 0.90 |
| Twin or triple: yes (%) | 3(3.7%) | 28(2.2%) | 0.63 |
| Feeding patterns |  |  | 0.35 |
| Exclusive breastfeeding | 36(45.0%) | 603(48.4%) |  |
| Partial breastfeeding | 39(48.8%) | 520(41.7%) |  |
| Formula feeding | 5(6.2%) | 124(9.9%) |  |
| Maternal working during pregnancy: yes (%) | 38(47.5%) | 576(46.2%) | 0.82 |
| Second-hand smoke exposure during pregnancy: yes (%) | 12(15.0%) | 163(13.1%) | 0.62 |

† AL/CR: ratio of axial length to corneal radius
