# Supplementary material for: Risk factors for astigmatic components and internal compensation: the Nanjing Eye Study
Source: Eye (Lond). 2020 Apr 22;35(2):499–507. doi: 10.1038/s41433-020-0881-5 (PMC8026993; doi:10.1038/s41433-020-0881-5)
Supplement: Supplementary file 3 — sTable 3 [file 41433_2020_881_MOESM3_ESM.docx]

**sTable 3. Distribution of Risk Factors in Children With and Without Oblique Internal Compensation**

| **Risk Factors** | **Without Oblique Internal Compensation (CF_45_) (N = 344)** | **With Oblique Internal Compensation (CF_45_) (N = 983)** | ***P*-value** |
| --- | --- | --- | --- |
| Mean (± SD) age (month) | 66.70±3.29 | 66.89±3.43 | 0.38 |
| Mean (± SD) paternal age at child birth (year) | 27.79±4.69 | 27.96±4.81 | 0.58 |
| Mean (± SD) maternal age at child birth (year) | 26.33±3.71 | 26.20±3.99 | 0.56 |
| Mean (± SD) birth weight (kilogram) | 3.34±0.53 | 3.33±0.52 | 0.67 |
| Mean (± SD) near-work activity (hour) | 4.67±3.38 | 4.79±3.64 | 0.58 |
| Mean (± SD) mid-working distance activity (hour) | 1.44±1.30 | 1.56±1.77 | 0.21 |
| Mean (± SD) outdoor activity (hour) | 2.06±1.02 | 2.28±1.45 | 0.002 |
| Mean (± SD) average nighttime sleep on weekdays (hour) | 9.91±0.67 | 9.89±0.67 | 0.53 |
| Mean (± SD) average nighttime sleep on weekends (hour) | 10.30±1.05 | 10.16±0.77 | 0.03 |
| †AL/CR | 2.87±0.07 | 2.87±0.07 | 0.83 |
| Gender: male (%) | 192(55.8%) | 514(52.3%) | 0.26 |
| Paternal myopia: yes(%) | 125(36.3%) | 354(36.0%) | 0.91 |
| Maternal myopia:yes (%) | 135(39.2%) | 394(40.1%) | 0.78 |
| Paternal astigmatism yes (%) | 51(14.8%) | 134(13.6%) | 0.58 |
| Maternal astigmatism: yes (%) | 59(17.2%) | 153(15.6%) | 0.49 |
| Mode of pregnancy: assisted (%) | 57(16.6%) | 168(17.1%) | 0.82 |
| Term delivery |  |  | 0.67 |
| Full-term | 311(90.4%) | 891(90.6%) |  |
| Pre-term | 15(4.4%) | 50(5.1%) |  |
| Post-term | 18(5.2%) | 42(4.3%) |  |
| 5-min Apgar score: Abnormal (%) | 10(2.9%) | 30(3.1%) | 0.89 |
| Delivery mode |  |  | 0.79 |
| Vaginal | 190(55.2%) | 559(56.9%) |  |
| Vaginal transferring to cesarean | 29(8.4%) | 73(7.4%) |  |
| Casarean | 125(36.3%) | 351(35.7%) |  |
| Oxygen uptake after birth: yes (%) | 21(6.1%) | 58(5.9%) | 0.89 |
| Second or third child: yes (%) | 72(20.9%) | 186(18.9%) | 0.42 |
| Twin or triple: yes (%) | 9(2.6%) | 22(2.2%) | 0.69 |
| Feeding patterns |  |  | 0.65 |
| Exclusive breastfeeding | 173(50.3%) | 466(47.4%) |  |
| Partial breastfeeding | 139(40.4%) | 420(42.7%) |  |
| Formula feeding | 32(9.3%) | 97(9.9%) |  |
| Maternal working during pregnancy: yes (%) | 152(44.5%) | 461(46.9%) | 0.44 |
| Second-hand smoke exposure during pregnancy: yes (%) | 51(14.8%) | 124(12.6%) | 0.30 |

† AL/CR: ratio of axial length to corneal radius
